# Nutritional regulation of sow reproduction by vitamin A: insights and knowledge gaps

**DOI:** 10.1186/s40813-026-00490-9

**Published:** 2026-02-19

**Authors:** Samantha R. Yankocy, Claire Stenhouse

**Affiliations:** Department of Animal Science, College of Agricultural Sciences, Pennsylvania State University, University Park, PA 16802 USA

**Keywords:** Pig, Pregnancy, Nutrition

## Abstract

Nutrition plays a critical role in maintaining overall animal health, productivity, and reproductive success. Among the key nutritional components essential for optimal physiological function are vitamins, organic compounds required in small amounts for metabolism and organism growth. One vitamin of particular importance in swine production is vitamin A due to its fundamental roles in numerous physiological processes, including the regulation of gene expression, maintenance of epithelial integrity, immune system function, and reproduction. This review synthesizes the current state of knowledge regarding the role of vitamin A in sow reproduction and aims to identify critical knowledge gaps that must be addressed to support the health, productivity, and longevity of the modern hyperprolific sow.

## Introduction

Over the past several decades, rapid genetic progress, refined management practices, and advancements in reproductive technologies have profoundly reshaped the productivity of commercial sow herds. Modern hyperprolific sows produce larger litters and sustain multiple high-output lactations across their productive lifespan. While these achievements have driven efficiency gains in global pork production, they have also imposed substantial metabolic and physiological demands on the breeding female. As a result, ensuring nutritional adequacy, not merely avoiding deficiency, has become a central focus in optimizing reproductive efficiency and lifetime productivity.

Among the key nutrients required for optimal physiological function are vitamins, organic compounds required in small quantities to support metabolism and growth. Traditionally recognized for its importance in vision and epithelial maintenance, vitamin A also serves as a regulatory molecule that influences gene transcription, cell differentiation, and morphogenesis through its active metabolites, notably retinoic acid. These metabolites function as ligands for nuclear receptors (retinoic acid and retinoid X receptors), thereby modulating the expression of genes essential to reproduction, embryogenesis, and immune competence [1].

Sow diets are typically formulated to meet or exceed the dietary requirements established by the National Research Council (NRC). According to the NRC (2012), the recommended daily intake of vitamin A is 8,398 IU for gestating sows and 11,932 IU for lactating sows [2]. Importantly, these values represent the minimum intakes required to prevent clinical deficiency, rather than concentrations designed to optimize reproductive performance and long-term health. In commercial production systems, several practical challenges further complicate the delivery of consistent vitamin A concentrations including substantial losses of retinyl acetate during feed processing and storage, which is not explicitly accounted for in the NRC requirement estimates [3]. In addition, the NRC recommendations are largely derived from data generated in highly controlled environments that may not fully reflect the variability and challenges encountered under on-farm management.

In recognition of these limitations, breeder nutrient specifications and commercial feeding programs commonly formulate sow diets with vitamin A concentrations substantially above the NRC suggestions, often in the range of ~ 10,000 IU/kg feed. These higher inclusion levels reflect industry efforts to ensure nutritional adequacy under commercial conditions rather than an intent to redefine the NRC requirements. Notably, the NRC recommendations for swine have not been updated since 2012 [2], and the experimental foundation for these values predates many recent advances in sow genetics and production efficiency. The modern sow has significantly increased reproductive performance, producing larger litters and experiencing greater physiological strain during gestation and lactation than earlier genetic lines, which may alter both vitamin A utilization and dietary requirements.

Accordingly, the key question is not whether NRC recommendations are sufficient to prevent deficiency, but rather what magnitude of vitamin A supplementation above NRC minimum is required to support reproductive performance and maternal health in contemporary hyperprolific sows. As reproductive capacity and metabolic demands continue to increase, re-evaluation of dietary vitamin A provision within modern genetic, physiological, and management contexts is warranted to ensure nutritional adequacy and optimize reproductive outcomes. This review synthesizes the current state of knowledge regarding the role of vitamin A in swine health and reproduction and aims to identify critical knowledge gaps that must be addressed to support the health, productivity, and longevity of the modern hyperprolific sow.

## Dietary sources of vitamin A

**Fig. 1:**
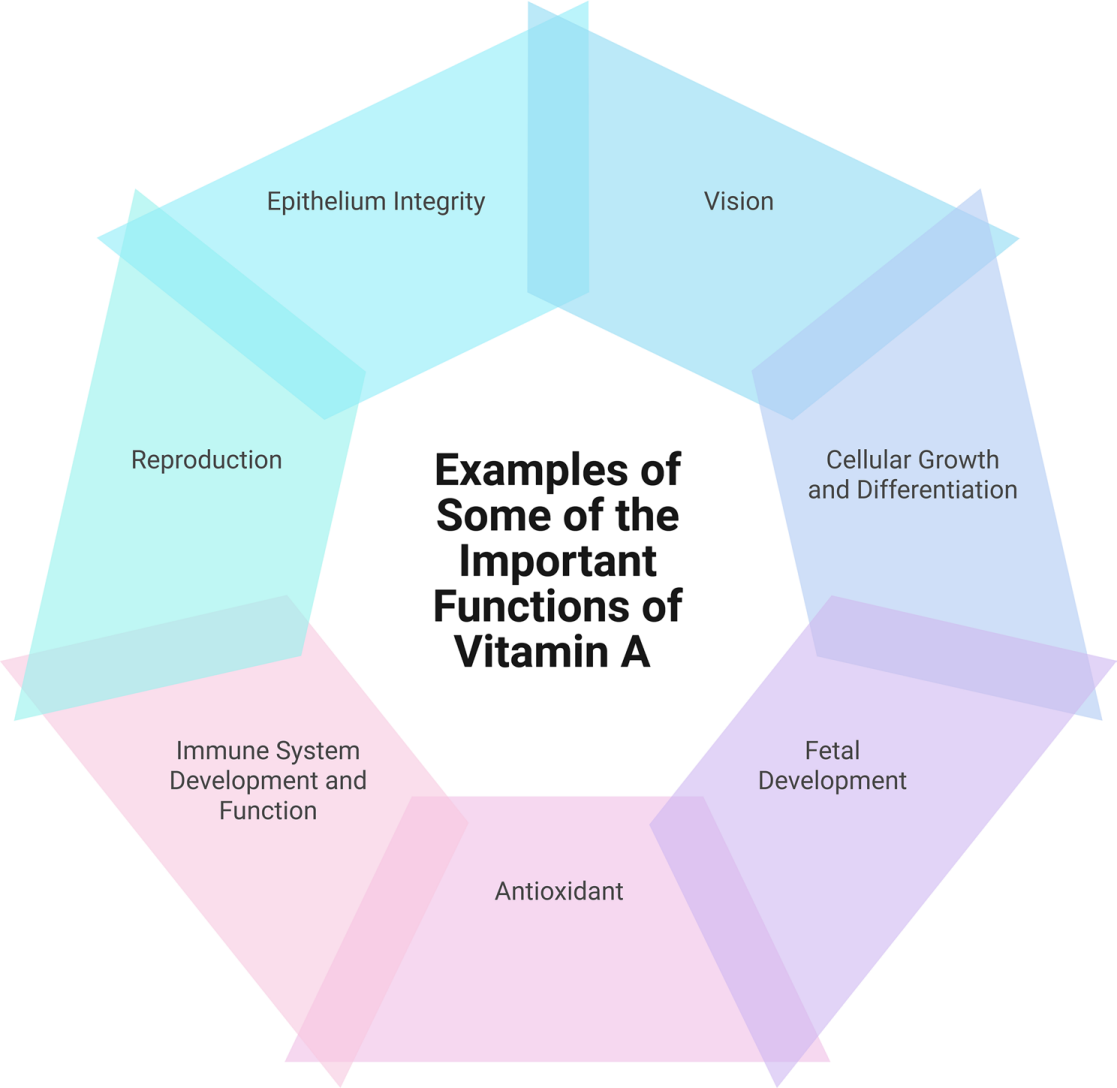
Summary of some of the essential roles of vitamin A for animal health and development. Figure generated using BioRender

Vitamin A, also known as retinol, is a lipid soluble vitamin with essential for a wide range of biological functions, including vision, growth, epithelial cell differentiation and proliferation, bone development, reproduction, and embryogenesis (Fig. 1). The term “vitamin A” encompasses a group of molecules termed retinoids: retinol (an alcohol), retinal (an aldehyde), and all-trans retinoic acid [4]. Meeting the vitamin A requirements of modern swine presents a considerable challenge due to contemporary housing and feeding practices prevalent in commercial production systems [5]. Historically, before the widespread adoption of full-confinement housing, swine consumed high-quality forages that were naturally rich in β-carotene [5]. In contrast, current swine diets are largely composed of cereal grains, primarily corn and soybean meal, which contain negligible amounts of vitamin A. While corn does contain β-carotene, the primary plant-based precursor of vitamin A, its concentration is low and its bioavailability to pigs is poor [6–8], making it an unreliable source to meet the vitamin A requirements of the animal [9].

β-Carotene must be enzymatically converted to retinol [10], a process not well characterized in pigs. Studies utilizing German Landrace x Piétrain pigs demonstrated that pigs convert β-carotene to retinol at a relatively inefficient rate of approximately 40:1, with variability influenced by the dietary ratio of β-carotene to vitamin A [11]. This rate is substantially lower than that observed in humans, whose conversion efficiency ranges from 3.6:1 to 28:1 depending on the food source [12]. Across mammalian species, it has been suggested that variation exists in the intestinal expression and activity of 15,15’-monooxygenase (BCMO1), the key enzyme responsible for the central cleavage of β-carotene into retinol [13, 14]. It has been suggested that BCO1 derived from pig has a molecular weight of 156 kDa [15] and is larger in size than comparable enzymes in other species [14], suggesting a potential difference in the activity of this enzyme in pigs. This, in combination with the impact of bioavailability of the dietary source of vitamin A, may contribute to the low rate of conversion observed in pigs. Given the limited availability of bioactive vitamin A in typical feedstuffs and the inefficient conversion of β-carotene, swine diets are commonly supplemented with preformed vitamin A in the form of retinyl esters, such as retinyl acetate or retinyl palmitate, which are absorbed with high efficiency (80–90%) [1, 9].

## Vitamin A metabolism

Vitamin A metabolism is a complex, multi-step process involving multiple enzymatic pathways and tightly regulated transport mechanisms in multiple organ systems. In swine, the primary source of vitamin A is ingested retinyl esters which are hydrolyzed by pancreatic lipases and small intestinal esterases to release retinol. Retinol can then be absorbed by the enterocytes, esterified, and incorporated into chylomicrons (large, spherical lipoprotein particles) for transport to the liver through the circulatory and lymphatic systems [16, 17]. The liver acts as a storage site for vitamin A, storing it in the form of retinyl esters in the stellate cells. When required, the retinyl esters can be hydrolyzed to free retinol, which binds to retinol-binding protein for systemic transport [18, 19]. Retinol binding protein (RBP) plays a key role in maintaining vitamin A homeostasis by facilitating its targeted delivery to tissues while preventing excessive accumulation and potential toxicity. In target tissues, retinol can be converted by enzymes such as alcohol dehydrogenases and retinal dehydrogenases to active derivatives that can regulate gene expression and cellular function in the target tissues. These biologically active forms of vitamin A binds to nuclear receptors, retinoic acid receptors (RARs) and retinoid X receptors (RXRs), which are widely expressed in cells in multiple organs including the liver, intestine, kidney, adipose, and immune system. The resulting receptor-ligand complexes modulate transcription by interacting with retinoic acid response elements in gene promoters [20], thereby influencing key physiological processes such as epithelial differentiation, immune function, vision, and metabolism.

## Vitamin A status and deficiency

It is well established that an animal’s nutritional requirements vary depending upon the stage of production and physiological stressors the animal experiences. Considering this, it is important for any nutrient to determine the reference levels for healthy animals at each stage of production; an area that has been poorly investigated in modern swine herds.

Serum retinol in weanling and nursery piglets have been recently reported. However, it is important to note that in many of these studies, the sows were intentionally fed diets deficient in vitamin A during gestation. Reported serum retinol concentrations in piglets vary depending on the study and whether supplementation has been performed but are approximately 5.70 µg/dL – 17.25 µg/dL between birth and day 42, depending on sow vitamin A status during gestation and whether supplemental vitamin A was provided postnatally [21, 22]. While serum retinol has recently been evaluated in young pigs, serum retinol remains under investigated in the modern sow. Previous reports indicate that serum retinol concentrations range from 12.5 to 25 µg/dL in Duroc, Yorkshire X Landrace X Hampshire, and German Landrace X Piétrain females [11, 23, 24]. Further, it has been reported that serum retinol concentrations in Duroc gilts at farrowing to be 20.0 µg/dL and 16.2 µg/dL in two different experiments when fed 6,500 IU and 7,100 IU of vitamin A (form not described) per day [23]. However, there remains significant gaps in knowledge surrounding how serum retinol concentrations change with parity or during gestation, and if these values are comparable in the modern hyperprolific sow that is housed in confinement with different feed sources.

Vitamin A deficiency leads to pathological changes in ocular tissues and the epithelial linings of the respiratory, reproductive, nervous, urinary, and digestive systems [25]. Swine deficient in vitamin A display clinical signs such as impaired weight gain, incoordination, hind limb paralysis, blindness, elevated cerebrospinal fluid pressure, and decreased vitamin A in both the circulation and liver stores [7, 26–30]. Vitamin A deficiency presents itself in pigs as reduced production efficiency in sows [31], increased embryonic mortality, and an increased number of stillborn piglets [24]. Deficiency during gestation may result in the birth of piglets that are blind, eyeless, weak, or exhibit congenital malformations [25]. A distinctive manifestation of maternal vitamin A deficiency is spinal cord herniation in fetal pigs. In growing pigs, deficiency is associated with impaired coordination, the onset of night blindness, and increased incidence of respiratory disease [25]. Once clinical deficiency is observed, the pigs have consumed a diet deficient in vitamin A for an extended period as the liver can store large amounts of vitamin A for a long time [23]. It has been reported that when mature gilts are fed a diet deficient in vitamin A, they will have three healthy litters before they exhibit clinical symptoms of deficiency [7]. Presumably, this prolonged period without exhibiting clinical symptoms of deficiency may be due to the significant storage of vitamin A (retinol and retinyl esters) in the liver [32, 33] which could be mobilized over an extended period of time prior to observing a decrease in serum retinol or clinical symptoms of deficiency. It is important to note that there is a gap in knowledge surrounding how long hepatic vitamin A stores sustain “normal physiology” under total dietary vitamin A deprivation, both in non-pregnant animals or in animals that are pregnant or lactating and have elevated nutritional demands. When evaluated in nursery Hampshire and cross bred piglets, serum retinol concentrations displayed values associated with deficiency after only 8 weeks when fed a diet with no vitamin A [34]. The placental transfer of vitamin A is poor in the pig and therefore the piglets must obtain a significant amount of vitamin A from colostrum and milk. In fact, it has been suggested that vitamin A is 30% lower in neonatal Yorkshire X Landrace piglets compared to their dam immediately before parturition [35]. Thus, the piglet will be reliant upon milk during the preweaning stage to allow the accumulation of significant vitamin A reserves that could be mobilized in instances of deficiency. Additionally, as nursery pigs have rapid growth and metabolic rates, their vitamin A requirement per kg of body weight is much greater than adults [29, 36, 37] therefore they may deplete their reserves of vitamin A much faster than adult animals. Considering this, it is important to determine the concentration of circulating retinol as well as the hepatic stores in healthy animals at each stage of production in modern swine herds. This information could help provide producers with valuable information related to the health of their animals and nutritional plan, thereby optimizing productivity and animal health.

## Effects of vitamin A on reproduction

There is a well-characterized link between reproductive efficiency and nutrition across livestock species. Importantly, vitamin A has been suggested to be important in the regulation of many essential processes in the female reproductive tract. The biological actions are mediated through binding to nuclear receptors, which modulates the expression of many genes in reproductive tissues integral to essential reproductive processes. In the ovary, retinol and retinyl esters are found in porcine corpora lutea and follicular fluid, with a strong correlation between the concentration of retinol in plasma and follicular fluid in cross bred females [38]. Cell specific localization of molecules with important roles in the regulation of retinol availability such as RBP, cellular retinoic acid-binding protein I (CRABPI) and retinoid X receptor β (RXRβ) proteins has been performed in the ovary and oviduct of cyclic sows [39]. RBP is not expressed by the ovary or oviduct of the cyclic sow [39] which suggests that perhaps these tissues do not utilize secretion of RBP-bound retinol into follicular or oviductal fluid. Instead, it could be speculated that the retinol present in the follicular fluid comes directly from the circulation. However, other molecules with key roles in the regulation of retinol availability are expressed in the ovary and oviduct. CRABP1 protein localized to the cytoplasm of large luteal cells and had apical localization in both ciliated and non-ciliated cells of the oviduct [39]. Similarly, RXRβ localized to both ciliated and non-ciliated cells in the oviduct and to the ovarian germinal epithelium and granulosa cells of cyclic sows, with an apparent increase in immunoreactivity in the granulosa cells from mature antral follicles compared to earlier stages of folliculogenesis [39]. It has been suggested in several mammalian species that retinoic acid signaling has an essential role in folliculogenesis by stimulation of proliferation and steroidogenesis in granulosa cells [40–43], suggesting a key role of vitamin A in the regulation of ovarian function.

In addition to roles in the ovary, retinol and retinol binding proteins have been extensively investigated in the uterus, with proposed roles of signaling through retinoic acid receptors for the regulation of uterine receptivity and decidualization [44]. In the pig, both retinol and RBP are highly abundant in uterine secretions, increasing during the luteal phase of the cycle and in early pregnancy [45–47]. Additionally, the porcine conceptus (embryo/fetus and associated placental membranes) in cross bred sows begins synthesizing and secreting retinol-binding protein (RBP) as early as Day 10 of gestation [48]. Collectively these findings suggest a significant role of vitamin A in the regulation of conceptus development.

During early gestation (days 14–30) in cross bred females it has been demonstrated that vitamin A is primarily present as retinol in the endometrium whereas in the myometrium it is stored mainly as retinyl esters, predominantly retinyl palmitate [49]. During this period, there is greater vitamin A in the endometrium compared to the myometrium, which may reflect the importance of vitamin A in the regulation of endometrial function during early pregnancy. Immunolocalization demonstrated that RBP is absent in the myometrium during this period of gestation and instead is localized to the glandular and luminal epithelium of the endometrium [49]. Retinol binding protein itself has been shown to have a significant role in the regulation of endometrial cell function, with RBP4 regulating proliferation, migration and apoptosis of endometrial epithelial cells and endometrial stromal cells to affect embryo implantation in the sow [50]. CRABP1 was also absent from the myometrium and localized to the cytoplasm of the glandular and luminal epithelium throughout early gestation, with apical localization in the uterine glands from Days 16–20 in cross bred females [49]. Uterine glands are essential uterine structures that function to secrete nutrients into the uterine lumen for utilization by the conceptus therefore the apical localization may suggest a key role of CRABP1 in the regulation of retinol availability for utilization by the uterine glandular epithelia or secretion as a component of uterine histotroph.

Importantly, it has been demonstrated that changes in estradiol and progesterone concentrations regulate retinol availability, suggesting that vitamin A may have a key role in the regulation of ovulation, oocyte and embryo transport, and the establishment and maintenance of pregnancy in swine. During the porcine estrous cycle, circulating concentrations of estradiol and progesterone exhibit distinct, complementary patterns (Reviewed by [51]). Estradiol rises during the late follicular phase, peaking just before ovulation, reflecting the growth and maturation of the dominant ovarian follicles. This peak in estradiol production by the dominant follicles triggers behavioral estrus and facilitates the preovulatory luteinizing hormone surge. Following ovulation, the corpus luteum forms and secretes increasing amounts of progesterone, which dominates the luteal phase. Progesterone concentrations remain elevated to induce changes to the endometrium to support potential implantation and then decline rapidly due to luteolysis if the maternal recognition of pregnancy signaling process does not occur, allowing the next cycle to begin. It has been demonstrated that progesterone increases the production of RBP by the porcine uterus, with greater secretion of RBP present in response to progesterone treatment in ovariectomized gilts or naturally during the luteal phase and early pregnancy [45–47].

In cyclic sows, CRABP1 is first detected in the oviduct on day 2 of the estrous cycle [39]. In contrast, strong immunoreactivity for RXRβ protein was present in the oviduct during pro-estrus and estrus, periods associated with high estradiol concentrations, with weak staining present during metestrus and diestrus, periods associated with low estradiol concentrations. In the uterus, RBP and CRABP1 immunolocalized to both the uterine glandular and luminal epithelia only during the diestrus stage of the estrous cycle [39], which corresponds to the period where there is sustained progesterone production for the corpora lutea. Similarly, there was an apparent increase in RXRβ protein immunoreactivity in the uterus after ovulation, with the greatest immunoreactivity present during diestrus and the lowest immunoreactivity present during proestrus. In contrast, RXRβ immunoreactivity in the myometrium was unaffected by stage of the estrous cycle [39]. Collectively, these studies demonstrate spatio-temporal expression of molecules with important roles in the regulation of retinol availability in the sow reproductive tract that is regulated by steroid hormones.

## Effects of vitamin A supplementation on reproductive outcomes

Optimal vitamin A supplementation is defined as the amount of vitamin A that supports maximal growth, feed efficiency, overall health, and reproductive function while ensuring adequate hepatic reserves [5]. Raw materials used in commercial swine diets typically contain low amounts of bioavailable preformed vitamin A, making supplementation necessary. Dietary retinyl esters (retinyl acetate or palmitate) are well absorbed (80–90%) whereas and injectable preparations offer targeted delivery [9]. However, in the United States, only multivitamin fat-soluble injectable formulations are commercially available. Additionally, there remains significant gaps in knowledge surrounding how long serum retinol concentrations remain elevated above baseline for in response to dietary or injectable vitamin A supplementation, their half-life, or how supplementation influences the storage of vitamin A by the liver.

There is conflicting evidence in the literature regarding the effects of supplemental vitamin A on reproductive performance in sows. Some studies report beneficial effects including decreased numbers of stillborn piglets and increased litter size, and others report no improvement. Retinyl palmitate (1 × 10^6^ IU; intramuscular) was administered to Landrace Large White X Duroc Hampshire gilts on day 15 after exhibiting their second estrus prior to mating during the third estrus [52]. Whaley et al. [52] demonstrated that vitamin A treatment tended to increase embryo number and survival, reduced the variation in embryonic size, and increased the average diameter of the embryos while having no effect on the number of corpora lutea or aromatase activity in the conceptus, suggesting that perhaps targeted vitamin A supplementation prior to breeding may improve pregnancy outcomes in gilts. Further, supplementation of the combination of vitamins A and D (312,000 IU vitamin A, 52,500 IU vitamin D) on day 85 of gestation to Yorkshire or Yorkshire cross bred sows resulted in fewer stillborn piglets compared to control piglets [53].

In contrast, it has been suggested that supplementation of prepubertal cross bred (Landrace [1/4], Yorkshire [1/4], Chester White [1/4], Large White [1/4]) gilts with retinyl palmitate (14,000 IU/kg/wk) from birth reduced myometrial area but did not alter other uterine wall components or uterine protein and retinol binding protein production during the pre-pubertal period [54]. After puberty and mating, treated gilts showed increased uterine length but no differences in uterine weight, number of fetuses, placental or fetal weights, or endometrial production of nondialyzable radioactivity, acid phosphatase, or retinol binding protein on days 44–47 of gestation [54]. These results indicate that while prepubertal vitamin A treatment modestly influenced uterine morphology, it did not impact overall uterine capacity at the administered dose.

Coffey and Britt administered intramuscular injections of 0, 50, 100, or 200 mg of β-carotene to sows on the day of weaning and found no effect on weaning-to-estrus interval or repeat service rate [55]. However, a significant dose × parity interaction was observed: while primiparous sows showed no change in reproductive outcomes, multiparous sows exhibited an increase in the number of live-born piglets and a reduction in stillbirths. In a subsequent experiment, sows received either 200 mg of β-carotene or 50,000 IU of vitamin A via intramuscular injection at weaning, mating, and seven days post-mating. Both treatments resulted in an improved litter outcome, increasing the number of live-born piglets from 10.0 to 10.6 and reducing the incidence of stillbirths. Similarly, supplementation of either 250,000 or 500,000 IU retinol palmitate to multiparous sows (day of weaning from previous lactation) and gilts (on the day of initiation of estrus synchronization), and the day of breeding significantly increased litter size and piglet survival in parity 1 and 2 sows, with a linear increase observed in total, live-born, and weaned piglets across treatments [56]. In contrast, no significant effects were observed in older sows (parity 3–6). However, there was a decrease in piglet birth weight in response to vitamin A treatment in younger sows, likely due to larger litter sizes. Similarly, intramuscular injection of vitamin A (450,000 IU retinol palmitate) on the day of weaning or breeding increased litter size from 10.3 to 10.7 piglets born alive [57]. While there was no effect on the incidence of stillborn piglets in this study, it is important to note that the incidence of stillborn piglets was low in this study. These findings suggest that high-dose vitamin A supplementation may enhance reproductive performance in younger sows but not in older sows, suggesting parity specific differences in vitamin A requirements. In contrast, a single intramuscular injection of 1,000,000 IU vitamin A (use of retinol palmitate or retinol acetate not listed) on either the day of weaning of the sow’s previous litter, day of breeding, or day 2, 6, 10, 13, 19, 30, 70, or 110 of gestation resulted in no improvement to the number of stillborn piglets in PIC-C22 sows [58]. Similarly, Heaney et al. fed deficient Duroc gilts 16, 5, or 2.5 µg of retinyl palmitate/kg body weight and demonstrated no effect on litter size, birth weight, or survival rate [59]. It is important to note that modern sows are often considered hyperprolific, producing larger litters with a greater incidence of still born piglets and preweaning mortality than their predecessors. This does bring into question how biologically relevant these findings are for the modern sow and highlights a gap in knowledge that warrants further investigation. The amount of vitamin A supplemented in these studies vastly exceeds the current dietary requirements [2]. Excessive vitamin A intake (hypervitaminosis A) in swine is associated with adverse effects including hepatotoxicity, musculoskeletal, dermatological, and neurological symptoms [60, 61]. Although no clinical signs of hypervitaminosis A were reported in this study, these risks highlight the need for caution and a balanced assessment of all aspects of animal physiology when interpreting the potential benefits of such high-dose interventions.

To compare how serum retinol concentrations recover in gilts fed a diet low in vitamin A for an extended period of time, a study was conducted where Yorkshire X Landrace X Hampshire gilts were fed a depleted vitamin A diet for 5 weeks then subjected to one of 3 treatments: (1) continuation of the depleted diet; (2) fed a diet that meets the NRC recommendations; (3) given a weekly injection of 12,300 IU vitamin A and fed a deficient diet [24]. Gilts in the depleted diet and vitamin A sufficient diet had similar plasma retinol concentrations over the 5-week period (mean concentration of 25 µg/dL) whereas the injected group had significantly elevated plasma retinol concentrations over the 5-week period (mean concentration of 102.9 µg/dL) [24]. The impact of plasma retinol concentrations of over 100 µg/dL on gilt health warrants further investigation as those concentrations are much higher than any previously reported. Additionally, it is unknown how long plasma retinol concentrations will remain elevated above baseline for or what the impact of this increase is on storage of retinol by the liver. While circulating retinol can be considered an incomplete assessment of vitamin A status, concentrations of over 100 µg/dL in a gilt is remarkably high compared to previous reports. A third investigation found serum retinol concentration in gilts at farrowing to be 20.0 µg/dL and 16.2 µg/dL in two different experiments when fed 6,500 IU and 7,100 IU of vitamin A (form not mentioned) per day [23]. Similarly, quantification of serum retinol concentrations in sows across Pennsylvania reported concentrations ranging from 15 µg/dL to 75 µg/dL [62]. These papers report comparable circulating retinol concentrations in sows. However, given the significant changes in husbandry practices and physiological differences associated with the modern hyperprolific sow, these studies may not be representative of the circulatory retinol concentrations present in modern sows.

It has also been observed that when high concentrations of vitamin A to nursery pigs at concentrations of 1,100,000, 880,000, 660,000, or 440,000 IU of vitamin A per kg it will take 16, 17.5, 32 and 43 days, respectively, to experience hypervitaminosis [63]. However, pigs fed 220,000 IU of vitamin A did not experience any symptoms of toxicity after 8 weeks [63]. The concentrations fed in this study far exceeds the recommended 2,200 IU/kg that is recommended for pigs of that size [2], but they suggest that pigs can tolerate higher concentrations in the feed than currently recommended without facing hypervitaminosis. However, there remains a gap in knowledge surrounding vitamin A storage capacity and time to depletion in swine.

Another important consideration when assessing the impact of vitamin A supplementation on reproduction is the form of retinol provided. In a series of studies, the effects of supplementation of retinyl palmitate and retinyl propionate on reproductive parameters in Large White X Landrace females were compared [64]. The experiments involved administering 1,000,000 IU intramuscular injection of retinyl palmitate on day 16 of the estrous cycle, and a 500,000 IU intramuscular injection of retinyl propionate either the day before weaning, on the day of mating, or six days post-mating. Results demonstrated that retinyl palmitate positively influenced follicular development, potentially via enhancement of the steroidogenesis pathway. Conversely, retinyl propionate showed no significant impact on ovulation rate or corpora lutea formation. It was concluded that retinyl palmitate was more effective than retinyl propionate in enhancing reproductive traits in swine. However, this conclusion is complicated by the fact that retinyl propionate was administered at half the dosage of retinyl palmitate (500,000 IU vs. 1,000,000 IU) and that the timing of injections varied between treatments. Therefore, additional research is needed to clarify the effects of these retinyl esters on reproduction when administered intramuscularly.

As discussed, there have been several studies investigating the role of vitamin A in swine health and reproduction. However, it is important to acknowledge that many of these findings are > 10–20 years old. During this time, rapid genetic progress, refined management practices, and advancements in reproductive technologies have profoundly reshaped the productivity of commercial sow herds. We now have hyperprolific sows that produce larger litters and sustain multiple high-output lactations across their productive lifespan. While these achievements have driven efficiency gains in global pork production, they have also imposed substantial metabolic and physiological demands on the breeding female. Given the significant changes in physiology of the modern sow, this does raise the question of how physiologically relevant are these previous publications? There is a significant need to validate these previous findings in the modern sow to ensure that we comprehensively understand the nutritional requirements of these animals to be able to maximize animal health and productivity.

## Conclusion

**Fig. 2:**
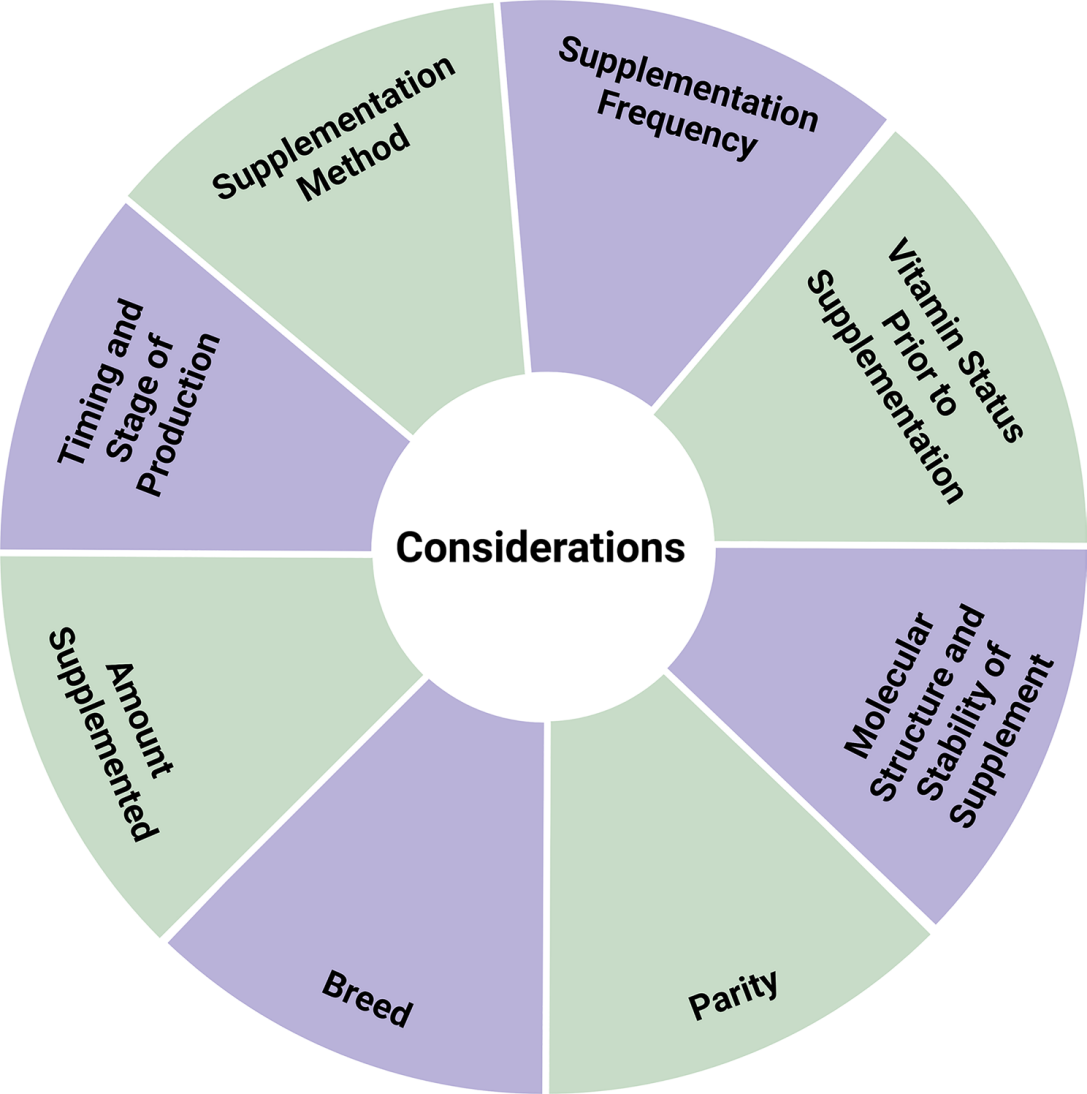
Considerations when supplementing swine with vitamin A. Figure generated using BioRender

Vitamin A is a nutrient that has been demonstrated to have essential roles in many aspects of sow health and productivity, including reproduction. As discussed, there are conflicting reports surrounding the effects of supplementation of vitamin A on reproduction, with some studies reporting beneficial effects such as decreased numbers of stillborn piglets and increased litter size, and others reporting no improvement; highlighting the need to consider multiple factors including the timing and stage of development when supplementation is provided, the form of retinol provided as a supplement, method of supplementation utilized, molecular structure and stability of the supplement, breed and parity of the sow, amount of supplement provided, and the vitamin status of the animal prior to supplementation (Fig. 2). Importantly, there have been minimal investigations into the nutritional requirements and physiological importance of vitamin A in the modern hyperprolific sow which requires further investigation.

## Data Availability

Not applicable. This article is a review manuscript and does not contain primary data; no datasets were generated or analyzed, and all relevant materials are available through the cited references.

## Abbreviations

CRABP1: Cellular retinoic acid-binding protein I
IU: International units
NRC: National research council
RBP: Retinol binding protein
RXRβ: Retinoid X receptorβ
